# Tongue cancer following hematopoietic cell transplantation for Fanconi anemia

**DOI:** 10.1007/s00784-022-04554-2

**Published:** 2022-05-28

**Authors:** Mattia Di Bartolomeo, Alexandre Anesi, Arrigo Pellacani, Sara Negrello, Annalisa Natale, Sabina Figurelli, Doriana Vaddinelli, Stefano Angelini, Luigi Chiarini, Riccardo Nocini, Paolo Di Bartolomeo

**Affiliations:** 1 Surgery, Dentistry, Maternity and Infant Department, Unit of Dentistry and Maxillo‐Facial Surgery, University of Verona, P.le L.A. Scuro 10, 37134 Verona, Italy; 2 Department of Medical and Surgical Sciences for Children & Adults, Cranio‐Maxillo‐Facial Surgery, University of Modena and Reggio Emilia, Largo del Pozzo 71, 41124 Modena, Italy; 3 Cranio-Maxillo-Facial Surgery Unit, University Hospital of Modena, 41124 Modena, Italy; 4 Department of Oncology Hematology, Intensive Care and Bone Marrow Transplantation, Ospedale Civile, Pescara, Italy; 5 U.O.C. Hematology and Cellular Therapy, Ospedale Mazzoni, Ascoli Piceno, Italy; 6 Section of Ear, Nose and Throat (ENT), Department of Surgical Sciences, Dentistry, Gynecology and Pediatrics, University of Verona, 37129 Verona, Italy

**Keywords:** Fanconi, Tongue, Squamous cell carcinoma, Oxidative stress, Bone marrow transplantation, Oral cancer

## Abstract

**Objectives:**

The aim of this retrospective study was to determine the incidence and the clinical outcome of tongue cancer (TC) in patients affected by Fanconi anemia (FA) who received an allogeneic hematopoietic cell transplantation (HCT).

**Materials and methods:**

The patient database from the Bone Marrow Transplant Center of Pescara was reviewed to enroll FA patients. Patients’, donors’, HCT’s, and screening’s data were collected as well to look for the incidence and the treatment of TC.

**Results:**

Twelve patients affected by FA were identified. Three patients died for transplant-related causes. Five of nine surviving patients were diagnosed with TC at a median of 21.7 years since transplantation and at a median age of 32.10 years. Interestingly, no patient manifested graft-versus-host-disease (GvHD). The 28-year cumulative incidence function of TC was 46.9% (95% CI, 36.9–56.9%). Two patients were treated with chemotherapy alone, two patients were treated with surgery alone, and one with surgery followed by chemotherapy. Overall, 4 patients with TC showed a clinical course characterized by a marked aggressiveness of the tumor disease which led to death due to cancer progression between 2 and 13 months. One patient is surviving 8 months after diagnosis of TC.

**Conclusions:**

Our study confirms the high incidence of tumors and in particular tongue tumors in allotransplanted FA patients. A careful screening has to be life-long maintained.

**Clinical relevance:**

Considering the rarity of FA and the frailty of FA patients, this study may add important information for the cancer management of these patients.

## Introduction

Fanconi anemia (FA) is a rare genetically and phenotypically heterogeneous autosomal and X-linked recessive disorder characterized by a broad spectrum of congenital malformations, progressive bone marrow failure (BMF), the presence of spontaneous and induced chromosomal breakage, and a marked predisposition to both hematological (myelodisplastic syndrome and acute myeloid leukemia) and solid tumors, specifically squamous cell carcinoma of head and neck (HNSCC) and genital region [1]. Hematological abnormalities occur in at least 90% of FA patients at a median onset of 7 years. Patients with FA have significantly increased cancer susceptibility because of defective DNA repair, which is a major molecular phenotype of the disease. The risk of head and neck squamous cell carcinoma is > 500-fold higher among individuals with FA compared with the general population [2, 3]. Allogeneic hematopoietic cell transplantation (HCT) is the only proven potential curative therapy and many adjustment have been made over time to improve HCT survival in FA patients [4, 5].

HCT is indeed able to completely and definitively correct BMF. Nonetheless, HCT is unable to eliminate the cancer susceptibility of FA patients and actually increases it considerably. The reason lies in the chemo-radiotherapic treatments used in the conditioning therapy and in the possible manifestation of graft-versus-host disease (GvHD) [6, 7].

Among HNSCC, in FA patients, the main role is played by oral cavity cancers [8]. In particular, tongue cancers have a rising incidence, representing almost 60% of all oral cancers in FA patients [2].

In this retrospective study, we describe the occurrence and the clinical outcome of tongue cancer (TC) in 12 FA patients who received an allogeneic HCT.

## Materials and methods

### Study cohort

Our study included 12 consecutive patients with FA who received an allogeneic HCT between May 1986 and October 2013 at the Bone Marrow Transplant Center of Pescara, Italy. The study was approved by the local institutional review board. Informed consent for the transplant was obtained from all patients and donors or their legal guardians in accordance with the Declaration of Helsinki.

### Data collection

Data were extracted from the Allogeneic Transplant Program Database of the Pescara Bone Marrow Transplant Center and included patient and donor demographic information (age, gender), hematologic disease diagnosis, donor relationship and HLA compatibility, stem cell source (either bone marrow, peripheral blood stem cells, or cord blood), drugs used in the conditioning regimen, and drugs used as GvHD prophylaxis. Other information regarding the post-transplant clinical outcome including engraftment, acute and chronic GvHD, survival, and most important complications were also obtained from the database. We examined both the incidence and the outcome of post-transplant TC.

### Patient characteristics

Baseline patient clinical characteristics are shown in Table 1. The median age at time of transplant was 11.9 years (range, 5.2 to 19.1). In all cases, the diagnosis of FA was made by the presence of pancytopenia both in the peripheral blood count and in bone marrow aspirate associated with typical physical malformations. The diagnosis was confirmed by demonstration of increased chromosomal breakage in patient lymphocytes by the addition of diepoxybutane [9]. Indication for HCT was bone marrow failure in all cases. No patient showed any sign of myelodysplastic syndrome or leukemic transformation at time of transplant. Positive family history of FA was present in 2 patients, who were brothers and had two other brothers who died for progressive marrow failure due to FA.

**Table 1:** Clinical characteristics of patients at time of HCT

						N. of transfusions		Peripheral blood counts		
UPN	Sex	Age years	Phenotypic anomalies	Interval diagnosis HCT months	Previous therapy	RBC	PLT	Hgb g/dL	PMN × 10.^9^/L	PLT × 10.^9^/L
073	M	13	Café-au-lait spots Short stature Osteoporosis Cryptorchidism Renal ectopy	8	None	4	0	7.1	450	68
082	M	11	Café-au-lait	27	None	2	0	8.8	1400	48
122	M	7	Short stature Hypoplastic thumb Cryptorchidism Clubfoot Single kidney	9	None	2	3	8.2	2400	29
183	F	13	Skin pigmentation Short stature Microcephaly Clinodactily	16	Androgens	0	0	8.7	1200	71
184	M	14	Café-au-lait spots Short stature Microcephaly	16	Androgens	1	0	8.5	1300	81
230	F	13	Café-au-lait spots Short stature Microcephaly Hearing loss	105	Androgens Steroids	3	0	8.2	650	61
328	M	6	Short stature Microcephaly Ear canal atresia Single palmar sulcus	18	None	1	0	8.6	900	27
333	F	11	Skin pigmentation Microcephaly Renal ectopy Horseshoe kidney Hearing loos	60	Androgens Steroids	0	0	7.9	2300	61
346	M	19	Café-au-lait spots	107	None	0	0	5.4	400	20
421	M	5	Café-au-lait spots Short stature	4	None	2	2	6.6	540	9
701	F	9	Facial dysmorphia Costal malformations Hypoplastic thumb Renal hypoplasia	25	None	0	0	8.3	880	22
973	F	12	Café-au-lait	91	None	1	0	10.9	700	32

Abbreviations: *HCT*, hemopoietic cell transplantation; *UPN*, unique patient number; *M*, male; *F*, female; *RBC*, red blood cell; *PLT*, platelet; *Hgb*, hemoglobin; *PMN*, polymorphonucleates

### Donor characteristics

Marrow donors were HLA genotypically identical siblings in 7 cases and 8/8 allele level HLA-matched unrelated donors in 3 cases. Two patients were transplanted with 5/6 allele HLA-matched unrelated umbilical cord blood (UCB) units. The donor median age was 19 years (range, 0 to 42). Five donors were males and 7 females.

### Transplant procedure

Each recipient was given unique patient number (UPN). The day of transplant was designated as day 0. The patients were treated in a laminar air flow room and maintained in a program of skin and mucosal decontamination. Ten patients received a preparative regimen consisting of low-dose cyclophosphamide (CY) (total dose 20 mg/kg) and total body irradiation (TBI) (total dose 5 Gy given on 3 fractions over 2 days), as previously described. One patient (UPN 706) received a combination of CY (50 mg/kg) and fludarabine (120 mg/m^2^) and one patient (UPN 973) was given a combination of thiotepa (5 mg/kg), busulfan i.v. (9.6 mg/kg) and fludarabine (120 mg/m^2^). For GvHD prophylaxis, 4 patients received cyclosporine A (CSA) alone and 8 were given a combination of CSA and short-course methotrexate. Hemopoietic stem cell source was bone marrow in 10 cases and UCB in 2.

### Definitions

Neutrophil engraftment was defined as the first of 3 consecutive days with an absolute neutrophil count ≥ 0.5 × 10^9^/L and platelet engraftment as the first of 3 consecutive days with an absolute platelet count ≥ 25 × 10^9^/L without transfusion support. Transplant-related mortality (TRM) was defined as all causes of death occurring at any time after HCT and not related to the underlying disease. Acute GvHD (aGvHD) was diagnosed according to Glucksberg’s criteria, and chronic GvHD (cGvHD) according to the modified Seattle criteria (for categorization of cGvHD as clinical limited or clinical extensive) [7, 10]. Patient delay represents the time in months between the date the first symptoms were noted by the patient and the date of treatment.

### Post-transplant cancer screening

In the first 5 years after transplantation, all patients were followed at least annually at the transplant center and every 2 to 3 years later or when a new clinical event appeared. They were constantly informed about the importance of an accurate screening for common cancers and in particular of cancer of oral cavity using brochures illustrating the increased risk of developing cancer.

Information on second tongue cancer, including date of diagnosis, site of involvement, morphologic features, therapy, and outcome, were collected by a review of medical records provided either by the patients or by the physician who made the diagnosis and had taken care of them. All cases were staged according to the American Joint Committee on Cancer (AJCC) staging system [11]. Pathology and physician reports of each case of STC were reviewed centrally at the transplant center by a committee including the transplant expert, the pathologist, the head and neck surgeon, and the oncologist. If necessary, tumors were reclassified in the clinical and pathological extension.

### Statistical analysis

Categorical and continuous data were summarized by median (range) and frequency, respectively. Taking into consideration death without occurrence of TC as competing risk, the probability of TC has been studied by fitting cumulative incidence function (CIF). Statistical analyses were performed with the use of R Statistical Software (version 3.3.3; R Foundation for Statistical Computing, Vienna, Austria).

## Results

### Transplantation

Eleven patients achieved full neutrophil and platelet engraftment after a median time of 14 days (range, 11–26) and 16 days (range, 11–35) after stem cell infusion, respectively. Acute GvHD affected 6 patients (grade I in 2, grade II in 2, and grade III in 2). Extensive cGvHD was diagnosed in 3 patients. Oral cGvHD was evident in 2 of them. Three patients (25%) died for transplant-related causes. One patient (UPN 346) died before engraftment on day 10 because of veno-occlusive disease of the liver, 1 patient (UPN 421) died on day 90 for aGvHD, and 1 patient (UPN 701) died on day 910 for cGvHD. One patient (UPN 073) died for accidental cause 8 years after transplantation. At time of data-censoring date (February 1, 2022), 3 patients out of 12 (25%) were living and doing well after 39 years (UPN 230), 25.6 years (UPN 328), and 8.1 years (UPN 973) after transplantation.

### Incidence and outcome of cancer after transplantation

**Fig. 1:**
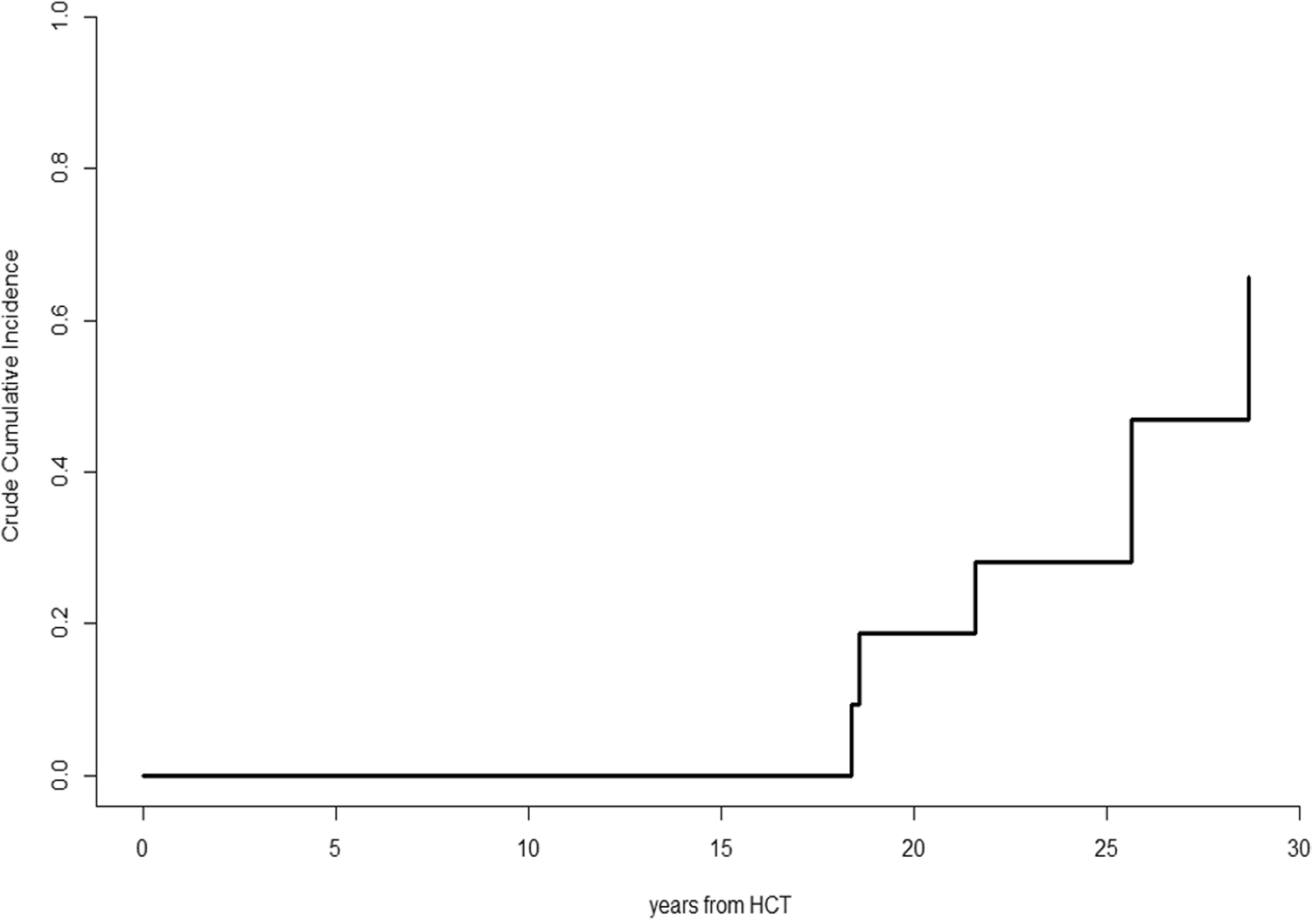
Cumulative incidence function of tongue cancers in Fanconi anemia patients.*HCT, hemopoietic cell transplantation

Five patients (2 males) developed TC at a median of 21.7 years (range, 18.4 to 28.8) since HCT. The 28-year CIF of TC was 46.9% (95% CI, 36.9–56.9%) (Fig. 1). Table 2 depicts details of patients who were diagnosed with TC. Of them, 3 had never been smokers and 2 had history of smoking and were smokers at time of TC. About alcohol consumption and drug abuse, only one patient was both moderate alcohol user and drug abuser at time of TC. No patient had ever shown signs of cGvHD and none was receiving systemic immunosuppressive therapy at time of. Histologic examination showed squamous cell carcinoma (SCC) in all cases. The median patient delay was 6 months (range, 0 to 24 months).

**Table 2:** Details of patients who were diagnosed with TC

UPN	Time to TC years	HT	CE	PE	HP grading	Stage	Patient delay months	Treatment	Status
082	18.4	SCC	T2N0Mx	pT2cN0cM0	G2	II	0	Surgery, CH	Progression, dead after 13 months
122	25.6	SCC	T2N2cM1		G3	IVC	24	CH	Progression, dead after 2 months
183	18.6	SCC	T2N2cM0		G3	IVA	6	CH	Progression, dead after 4 months
230	28.8	SCC	T2N0M0	pT1cN0cM0	G2	I	9	Surgery	Alive & well after 7 months
333	21.7	SCC	T3N0M0	pT3pN3bcM0	G2	IVb	5	Surgery	Progression, dead after 7 months

Abbreviations: *TC*, tongue cancer; *UPN*, unique patient number; *HT*, histologic type; *SCC*, squamous cell carcinoma; *CE*, clinical extension; *PE*, pathological extension; *HP*, hystopathologic; *CH*, chemotherapy

Two other patients were diagnosed with cancer other than TC. One male patient (UPN 328) was diagnosed with papillary thyroid carcinoma (T1N0M0) 12 years after transplantation for which he underwent total thyroidectomy and no other subsequent therapy. This patient was subsequently diagnosed with an ear skin melanoma 20 years after HCT which was radically excised with surgery. He is now in good health condition at 25 years since HCT with Karnofsky score of 100%. One patient (UPN 184) was diagnosed with SCC of the esophagus (cT2cN0cM0) at 21 years after HCT for which he was initially treated with radiotherapy followed by chemotherapy. One year later, the tumor disease reappeared in the same site with invasion of adjacent tissues and diffuse nodal metastases. The patient died 2 years after diagnosis of the cancer. To conclude, 1 patient (UPN 973) had a post-transplant outcome complicated by severe lung cGvHD. This patient is now living at 8 years after HCT without any immunosuppressive therapy but with a limited respiratory function and a Karnofsky score of 80%.

### TC case by case presentation

#### UPN 082

He presented to the outpatient clinic with an ulceration of the left lingual margin, with the histologic specimen confirming the diagnosis of a well-differentiated SCC. A left hemi-glossectomy was performed in a different surgical center, with clear surgical margins. No lymph node dissection and no adjuvant therapy were performed. Ten months after the surgery, the patient presented with a left cervical lymph node swelling. It was histologically diagnosed as a site of carcinoma recurrence. He underwent three courses of cisplatin and 5-fluorouracil-based chemotherapy. No tumor response was obtained, and the patient died 13 months since TC diagnosis.

#### UPN 122

The patient showed a painful swelling of the left lingual margin, but as a consequence of his severe anxious depressive state, he refused any diagnostic approach for 24 months. Finally, he agreed to undergo a biopsy of the lingual lesion, which documented a well-differentiated SCC. Unfortunately, the disease showed multiple lung metastases. He received a palliative treatment with low-dose chemotherapy and died 2 months later.

#### UPN 183

The patient, who was affected by severe mental retardation, showed a painful swelling of the right lingual margin. Six months since the first symptoms, the patient underwent a tumor biopsy that showed a moderately differentiated SCC. There was no data available regarding lymph node involvement. She received three courses of cisplatin and 5-fluorouracl-based chemotherapy, but no response was observed. The outcome was complicated by severe neutropenia followed by an idiopathic pneumonia which was the cause of death at 4 months since the TC diagnosis.

#### UPN 230

**Fig. 2:**
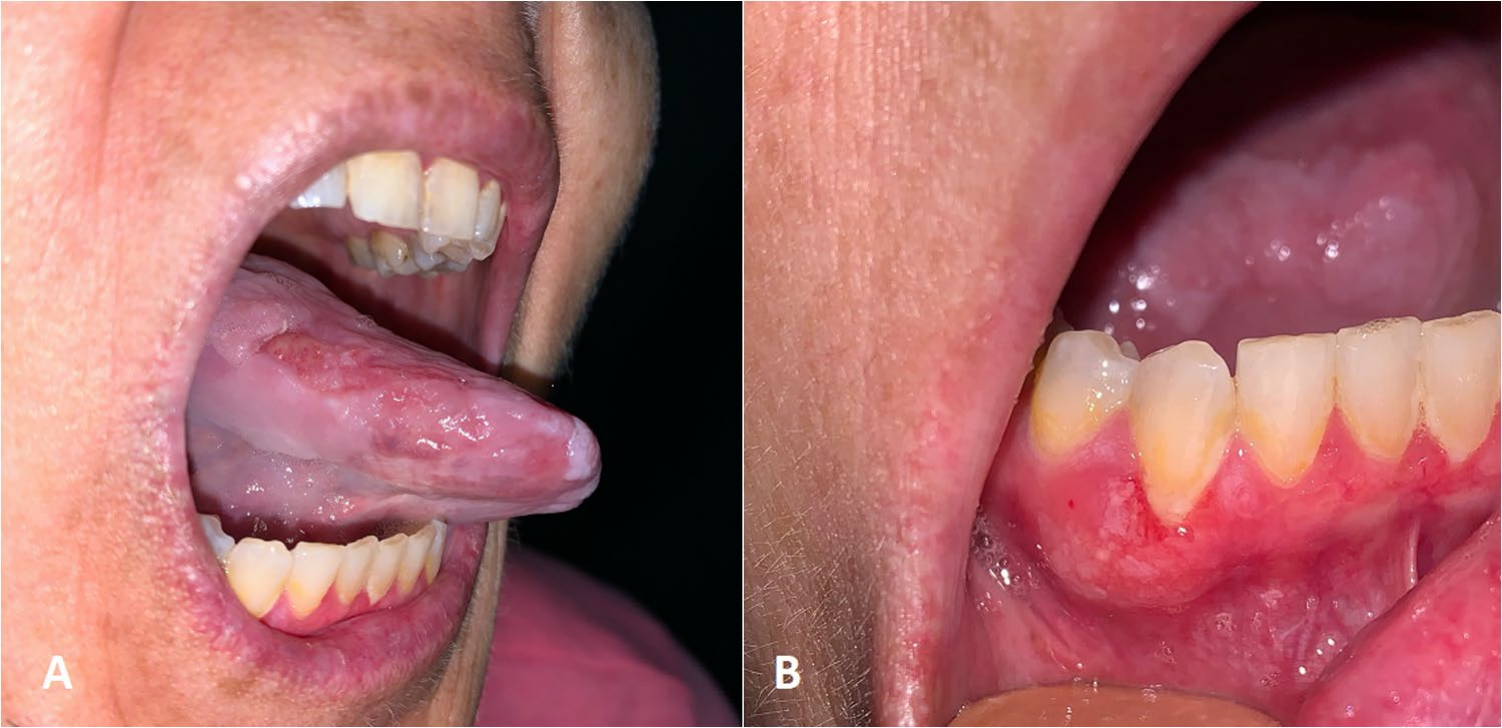
UPN 230 presented to the outpatient clinic with a painful swelling of the right side of the tongue (**A**). A concomitant ulceration of the adherent gingiva in the fourth quadrant was observed as well during the clinical examination (**B**). Bioptic examination of the lesions documented a squamous cell carcinoma in both cases

She presented to the outpatient clinic with a painful swelling of the right lingual margin. During the clinical examination, a concomitant ulceration of the adherent gingiva in the fourth quadrant was observed (Fig. 2). A prompt biopsy of both lesions led to the diagnosis of two moderately differentiated SCC. Preoperative radiological examinations showed no cervical nor distant metastasis. The patient underwent partial tongue resection and partial mandibulectomy, with clear surgical margins. The mandibular defect was contextually reconstructed with a right facial artery musculo-mucosal (FAMM) flap. The patient is now living and under strict clinical and radiological follow-up at 8 months post-operatively.

#### UPN 333

**Fig. 3:**
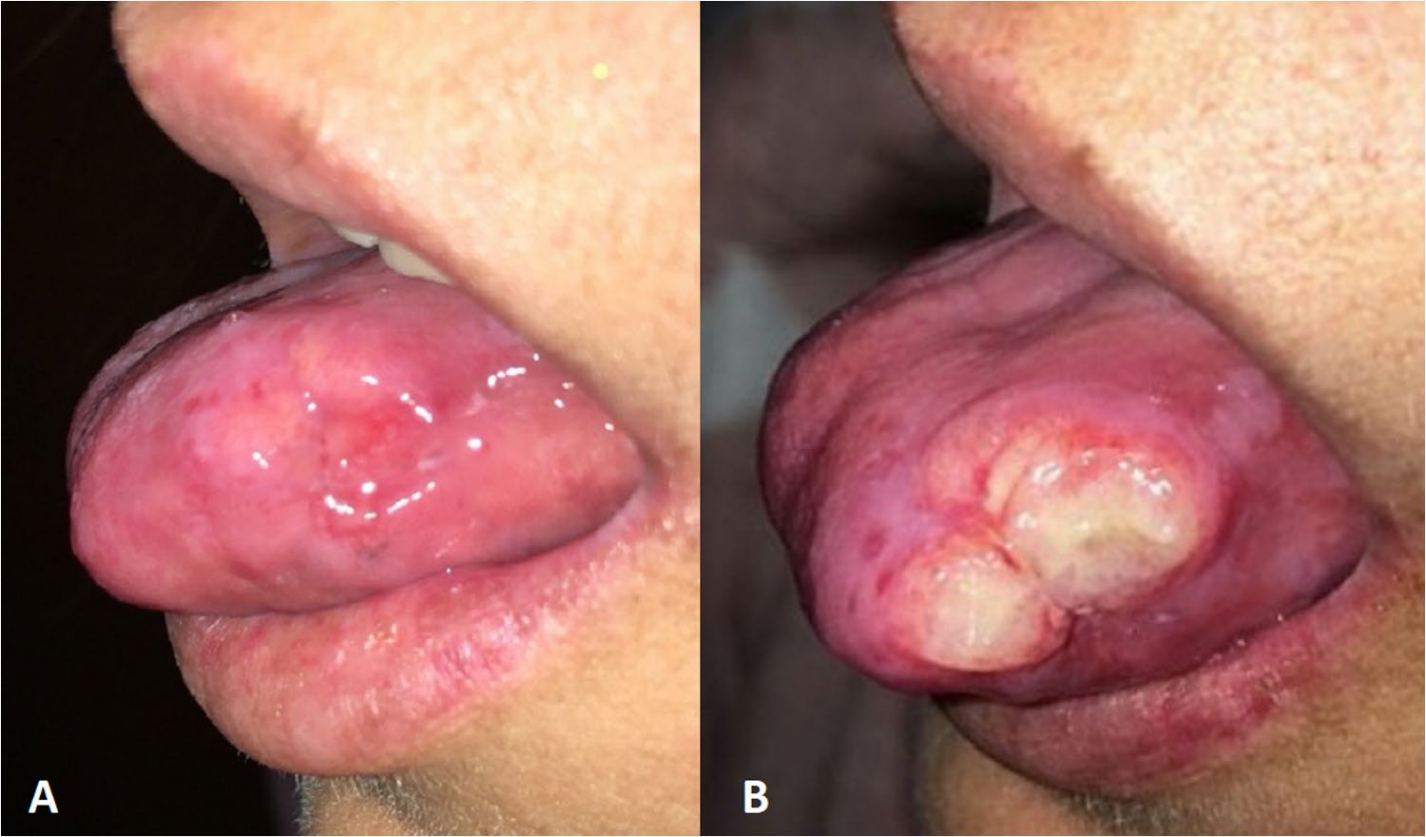
UPN 333 showed a painful swelling of the left lingual margin (**A**). The COVID-19 pandemic determined a 5-month diagnostic delay, with a consistent growth in size of the tumor (**B**)

The patient showed a painful swelling of the left lingual margin. Due to the actual COVID-19 pandemic, unluckily, a 5-month delay in tumor diagnosis was determined. During this time, the lingual swelling considerably grew in size (Fig. 3). Eventually, the patient underwent subtotal glossectomy with bilateral lymph node resection and tracheotomy. The histological result showed a moderately differentiated SCC with clear margins. Homolateral cervical lymph nodes were involved by tumor metastases. The poor general clinical conditions prevented any form of radiotherapy or chemotherapy and the patient died of cachexia 7 months after TC diagnosis.

## Discussion

FA patients are known to be more prone to developing malignant tumors. It is believed that this predisposition is linked both to the congenital instability of the chromosomes present in these patients and to a possible immunodeficiency. Fortunately, the optimization of HCT techniques has led to a lengthening of life expectancy in patients with FA. This resulted in an improvement in the immune capabilities of these patients. However, HCT opened the door to the long-term complications of this pathology at the same time.

For example, it was possible to observe an incredible predisposition to develop solid malignancies. In a recent study by Risitano et al., overall survival at 30 years was 37% and the main cause of death was represented by cancer [12]. In particular, HNSCC are very common in patients with FA. It is believed that they are 500 times more likely to develop HNSCC than the general population [2]. It also has to be underlined that the worst DNA damage can lead to an earlier development of malignancies. In the literature, it has been reported that the median onset age of FA-HNSCC is 32 years, compared to a median age of 63 years old in the general population [13, 14]. Several aspects have to be discussed to assess the reasons behind this increased risk.

First of all, the role of oxidative stress has to be cleared. In fact, it has been shown that oxidative stress can play a role in pathogenesis of various diseases, such as other oral conditions or in neurodegenerative diseases [15, 16]. The formation of oxygen radicals increases indeed the potential damage to the DNA. In FA patients, the ability to recover is limited, and therefore, the damage to DNA can potentially determine a malignant transformation of oral cavity cells. These intriguing aspects have led to hypothesize the central role of mitochondrial dysfunctions in FA patients [17, 18]. Animal models have been proven very useful in studying the underlying mechanisms of many conditions, included FA [19, 20]. In this case, chemoprevention therapies have been tested in vivo to reduce oxidative stress, with promising results [21].

Among the sites of the oral cavity, it should be noted that the tongue is affected in 60% of cases. In the general population, this data drops to 10–20%. The reason may lie in the high replication rate of tongue mucosal cells, subjected to a continuous mechanical stimulus due to movement. It has been estimated that gingival epithelium turnover time is 41 to 57 days, while the turnover time for cells of the dorsal surface of the tongue is around 5 days [22, 23].

The higher turnover rate creates a statistical increase in the possibility of replicative error, which is itself more likely in FA patients. Generally speaking, difficult oral care and extensive dental caries are frequent in patients with GvHD. So, the tongue is often subjected to traumatic frictional damage on sharp/rough teeth surfaces [24]. Repairing the DNA damage causes a potential increase in replicative error in this case as well. Nonetheless, it is worth to notice that oral tongue cancer incidence has been increasing worldwide [25, 26].

It therefore appears evident that an early and frequent screening is required in patients with FA, possibly by a multidisciplinary team. In fact, even constant dental monitoring could play a key role in the prevention of HNSCC. Unfortunately, the diagnostic delay in oral carcinomas is well documented [27]. Moreover, the present COVID-19 pandemic has increased the delays in oncologic diagnosis and treatments in the head and neck district [28]. As shown by Murillo et al., the onset of close cancer screening programs can lead to diagnose of SCC in stage I. Moreover, the follow-up has to be maintained lifelong because the cumulative incidence of SCC is up to 14% and 71% at 15 and 30 years, respectively [29].

Another key point in the pathophysiology of Fanconi anemia-related squamous cell carcinoma of head and neck (FA-HNSCC) is HCT. In order to grant a longer life expectancy and to prevent the development of leukemias, most FA patients undergo a HCT. Unluckily, patients treated with allogeneic HCT are at increased lifelong risk of developing malignancies. In the largest study to date to evaluate risk factors for solid cancers, Rizzo et al. studied a multi-institutional cohort of 28,874 allogeneic transplant recipients in which 189 solid malignancies were diagnosed over time. Overall, transplant recipients developed an invasive solid cancer at twice the rate expected based on population incidence rates. The risk reached threefold among patients followed for 15 years or more after transplantation. cGvHD and male sex were the main determinants for risk of malignancies. The most elevated excess absolute risk of developing solid cancer was observed for tumors of the oral cavity (overall 2.66 EAR, of which 1.08 tongue, 0.68 lip, 0.47 gum, and 0.43 salivary gland) [30]. GvHD is not present only in FA patients and it is one of the main risk factors for oral cancer [31, 32].

The therapeutic strategy is also very important in the management of HNSCC in patients affected by FA. The classic available weapons (surgery, radiotherapy, chemotherapy) must be well weighted. In fact, the predisposition to chromosomal instability of FA patients is related to an increased sensitivity to radiotherapy and chemotherapy [33], in which the resulting mucositis are really important, leading even to the interruption of the treatment. Furthermore, the poor DNA repair capacity of FA-affected cells does not guarantee a conspicuous replicative advantage over carcinoma cells. Therapies at lower doses or administered less frequently could be used, but with a consequent reduction in efficacy [34].

It is interesting to notice that those who present important collateral effects following radiotherapy or chemotherapy, especially young patients, should be genetically tested to exclude a FA-related mutation [35, 36].

Targeted chemo-therapy (such as cetuximab or gefitinib) or immuno-therapy (e.g., nivolumab, pembrolizumab) are definitely intriguing, but their actual efficacy and complication rates have still to be assessed overall [37]. It follows that the first-line therapeutic strategy is surgery. As demonstrated by Lee, patients with FA can also undergo major surgery without developing particular complications [37]. Conspicuous resective interventions are also possible, associated with reconstructive surgery which may include free flaps as well. However, the best strategy is to play in advance as in any cancer. The importance of frequent and early-onset screening is the key in the possibility of making diagnoses at an early stage [29]. So, the surgical resection of the tumor could be sufficient and effective as a treatment, possibly followed by reconstruction with local flaps. Careful preoperative planning must be performed in the treatment planning of any HNSCC, even with the aid of novel artificial intelligence tools, in order to ensure the preservation of noble structures [38, 39].

Another consideration should be made about local lymphatic surgery. From a therapeutic-diagnostic point of view, prophylactic nodal neck dissection in early (T1–T2) tongue cancers is still a controversial theme in non-FA patients [40, 41]. Namely, a prophylactic nodal neck dissection was not indicated in patient UPN 230, because the depth of invasion was less than or equal to 3 mm in all the specimens. As proposed by van Lanschot et al., a depth of invasion of at least 4 mm can be assumed as a key parameter to perform an elective node dissection in early stage oral cancer [42]. Moreover, it may be intentionally proposed to temporarily postpone nodal neck dissection in a FA-patient. In this way, an effective and predictable lymphatic drainage would be maintained in place. The card of nodal neck dissection could then be played later, given the predisposition to further HNSCC development in FA-patients. Nonetheless, a case-by-case approach should be adopted, together with a multidisciplinary board discussion.

In spite of progression in research and therapy, the prognosis still remains poor in FA-HNSCC patients, with about 50% mortality due to tumor progression in the first 2 to 3 years [12]. Considering the necessity of a close follow-up, adequate clinical and instrumental examinations are both required. Despite the sensitive improvement in radiation exposure when performing computed tomography scans, this aspect should also be taken in consideration in FA patients. Magnetic resonance imaging should therefore be preferred, especially when soft tissues have to be investigated. As already specified, close dental re-evaluations should also be performed, looking for potential cancer threats [43, 44].

## Conclusions

This study has identified five allotransplanted patients affected by Fanconi anemia with tongue cancer. The results of this paper indicate that TC can develop in a young age in this cohort of patients, even in absence of cGvHD. The precise mechanism of TC development remains to be elucidated, but key roles might be played by the oxidative stress, the short turnover time of tongue cells, and HCT. A close oral follow-up has to begin early and lifelong maintained, in order to detect and treat cancers with surgery at an early stage.
